# Whole-Genome Sequence Analysis of Shiga Toxin-Producing *Escherichia coli* Isolated from Livestock Animals in Ghana

**DOI:** 10.3390/microorganisms14010212

**Published:** 2026-01-16

**Authors:** Yusuke Ota, Samiratu Mahazu, Ivy Brago Amanor, Frederick Ofosu Appiah, Jennifer Amedior, Emmanuel Darko, Mitsunori Yoshida, Masato Suzuki, Yoshihiko Hoshino, Toshihiko Suzuki, Anthony Ablordey, Ryoichi Saito

**Affiliations:** 1 Department of Molecular Microbiology and Immunology, Graduate School of Medical and Dental Science, Institute of Science Tokyo, Tokyo 113-8510, Japan; y-ota.micr@tmd.ac.jp (Y.O.);; 2 Department of Parasitology and Tropical Medicine, Graduate School of Medical and Dental Science, Institute of Science Tokyo, Tokyo 113-8510, Japan; 3 Department of Bacteriology, Noguchi Memorial Institute for Medical Research, Accra P.O. Box LG43, Ghana; 4 Department of Mycobacteriology, Leprosy Research Center, National Institute of Infectious Diseases, Tokyo 162-8640, Japan; 5 Antimicrobial Resistance Research Center, National Institute of Infectious Diseases, Tokyo 162-8640, Japan; 6 Department of Bacterial Pathogenesis, Infection and Host Response, Graduate School of Medical and Dental Sciences, Institute of Science Tokyo, Tokyo 113-8510, Japan

**Keywords:** Shiga toxin-producing *Escherichia coli*, whole-genome sequence analysis, livestock animals, O38:H26, O43:H2, O157:H7

## Abstract

Shiga toxin-producing *Escherichia coli* (STEC) is a zoonotic pathogen of public health concern, requiring a One Health approach to clarify its transmission and distribution. However, its prevalence and genomic characteristics in livestock and companion animals remain underexplored in low-income countries. We investigated prevalence and genomic features of STEC in animals in western Ghana, representing the first genomic report of STEC in Ghana. Fecal samples (97) were collected from goats (*n* = 33), sheep (*n* = 33), dogs (*n* = 30), and a cat (*n* = 1), with STEC detected in 12.1% of goats and sheep samples. Whole-genome sequencing identified serotypes O38:H26, O43:H2, and O157:H7. *stx1c* and *stx2b* genes were detected in O38:H26 and O43:H2, whereas *stx2c* and key virulence genes (*chuA*, *eae*, *esp*, *nle*, *tir*, and *toxB*) were exclusively found in O157:H7. Phylogenetic analysis revealed that O38:H26 isolates form a cluster closely related to clinical strains from the UK. O43:H2 isolates exhibited diverse *stx* profiles, linking animal, environmental, and clinical strains from North America and the UK. O157:H7 isolates were genetically similar to European clinical and food-derived strains, suggesting that goats and sheep are important STEC reservoirs in Ghana, offering data for public health risk assessment and effective One Health-based control strategies.

## 1. Introduction

Diarrheagenic *Escherichia coli* is a major etiological agent of gastrointestinal diseases, representing a substantial public health concern due to its capacity to cause dehydration and other severe complications. In a previous study, we identified multiple diarrheagenic *Escherichia coli* pathotypes, including the highly virulent Shiga toxin-producing *E. coli* (STEC), among outpatients under the age of five in Ghana, underscoring the need for expanded epidemiological surveillance [1]. STEC is a major zoonotic pathogen with a complex ecology encompassing human, animal, and environmental reservoirs [2]. It is responsible for severe enteric diseases, including hemorrhagic colitis and hemolytic uremic syndrome (HUS), with an estimated global burden of 2.8 million infections annually, resulting in approximately 3890 HUS cases and 230 deaths [3]. Given the widespread occurrence of STEC in livestock, wildlife, and environmental sources, a One Health approach, which integrates human, animal, and environmental health, is essential for understanding and mitigating its transmission dynamics. Cattle are the primary reservoir of STEC and a major source of human infection due to their high carriage rates and their role in food production [4]. Livestock and companion animals, particularly small domestic ruminants such as goats and sheep, are also key reservoirs of STEC due to their close interactions with humans [5,6]. Transmission to humans occurs through direct contact with these animals or through ingestion of contaminated water, meat, and dairy products, emphasizing the importance of targeted surveillance and control measures to elucidate reservoir status and characterize STEC isolates [7,8,9].

STEC strains vary in their virulence gene profiles, influencing their pathogenicity and ability to cause severe human diseases [6,10]. Among these genes, Shiga toxin (Stx), a key virulence factor of STEC comprising *stx1* and *stx2*, plays a pivotal role in disease pathogenesis. Subtypes *stx2a*, *stx2c*, and *stx2d* have been linked to severe clinical outcomes (5). STEC also comprises both O157 and non-O157 serotypes, which differ in their epidemiological and ecological characteristics, including infection prevalence and host carriage rates [11,12]. A random-effects meta-analysis estimated the pooled prevalence of *E. coli* O157 in cattle to be 7.35% in North America, 6.85% in Oceania, 5.15% in Europe, 4.69% in Asia, and 1.65% in Latin America and the Caribbean [13]. In Africa, One Health-based studies reported a pooled prevalence of *E. coli* O157 at 4.7%, with the highest rates found in animal samples (5.4%), followed by environmental (3.4%) and human samples (2.8%) [14]. Serotype O157 is most commonly associated with severe diseases, such as hemorrhagic colitis and HUS, whereas non-O157 serotypes can also cause diarrheal illness and, in some instances, HUS. Phylogenetic analyses are crucial for identifying region-specific types and tracking dissemination patterns to inform outbreak prevention [15]. In Africa, STEC is a notable public health concern, with genomic investigations from countries such as Ethiopia, Kenya, Nigeria, and South Africa revealing diverse serotypes and genetic profiles in livestock, underscoring the importance of whole-genome sequencing for understanding its regional strain diversity [16,17,18,19]. However, in Ghana, the epidemiology of STEC remains poorly understood, with limited data on its prevalence in livestock and a lack of genomic characterization of local isolates, primarily due to the absence of surveillance systems targeting non-clinical strains.

This study, therefore, aimed to investigate the prevalence and genetic characteristics of STEC in non-bovine livestock and companion animals in the western region of Ghana, a region that reflects the resource constraints typical of low-income settings. Whole-genome data from STEC isolates provides crucial insights into their virulence potential, phylogenetic relationships, and public health risks, addressing a major knowledge gap and informing evidence-based surveillance and control strategies in the region.

## 2. Materials and Methods

### 2.1. Sample Collection and STEC Isolation

A total of 97 rectal swab samples of livestock and companion animals were used in the study. The samples originated from goats (*n* = 33), sheep (*n* = 33), dogs (*n* = 30), and a cat (*n* = 1). The samples were obtained from the Sekondi-Takoradi Veterinary Services Department of Ghana and were collected from 14 August to 20 September 2023. The livestock and companion animals were maintained in household or farm settings in Sekondi-Takoradi Metropolis. Rectal swabs were carefully taken by veterinary technicians with little or no discomfort to the animals. The samples were put into Cary–Blair transport medium, transported in a cold box to the laboratory, and stored at −80 °C until further analysis. STEC were isolated using CHROMagar STEC (CHROMagar Microbiology, Paris, France) following the manufacturer’s instructions [20]. Presumptive STEC colonies, identified by their characteristic coloration on the chromogenic agar, were subcultured onto sheep blood agar plates and incubated at 37 °C for 18–24 h. Next, the bacterial species of the isolates were determined via 16S rRNA gene sequencing [21]. Briefly, the DNA of each bacterium was extracted using the Cica Geneus DNA extraction kit (Kanto Chemical, Tokyo, Japan). 16S rRNA genes were amplified using EmeraldAmp^®^ MAX PCR Master Mix (Takara Bio Inc., Shiga, Japan), and PCR products were purified using ExoSAP-IT^®^ (Thermo Fisher Scientific, Waltham, MA, USA). Targeted DNA sequencing was performed on a 3730xl DNA Analyzer (Thermo Fisher Scientific) using the BigDye Terminator v3.1 Cycle Sequencing Kit and BigDye XTerminator Purification Kit (Thermo Fisher Scientific). The sequences were queried against the Ribosomal Database Project for species identification based on the closest match. Detection of *stx1* and *stx2* genes in the isolates was conducted using Polymerase Chain Reaction (PCR) with multiple primer sets as described by Müller et al. [22] and Fujioka et al., with variant detection including *stx1*_OX3_, *stx1*_CB_, *stx2c*, *stx2d* (*stx2d-O111*, *stx2d-oxa*, *stx2d-ount*, *stx2d-NV206*, and activatable *stx2d*), *stx2e*, and *stx2g* [23]. The amplified DNA was electrophoresed on an agarose gel (PrimeGel™ Agarose LE 1-20K, Takara, Shiga, Japan) and band patterns were visualized using a UV transilluminator (ATTO, Tokyo, Japan) after ethidium bromide staining.

### 2.2. Antimicrobial Susceptibility Testing

Antimicrobial susceptibility testing was performed using the broth microdilution method with a MicroScan panel (Beckman Coulter, Brea, CA, USA) following the Clinical and Laboratory Standards Institute (CLSI) M100-S32 [24]. The antimicrobial agents tested included ampicillin, ampicillin–sulbactam, ceftolozane–tazobactam, piperacillin–tazobactam, cefazolin, cefepime, ceftriaxone, ceftazidime, cefmetazole, cefpodoxime, cefpodoxime–clavulanate, cefditoren, flomoxef, latamoxef, aztreonam, meropenem, faropenem, colistin, gentamicin, amikacin, minocycline, levofloxacin, trimethoprim–sulfamethoxazole, fosfomycin, and tigecycline. The minimum inhibitory concentrations were determined using the MicroScan AutoSCAN4 system (Beckman Coulter), and the results were interpreted as susceptible, intermediate, or resistant according to the CLSI M100-S32 breakpoints [24].

### 2.3. Whole-Genome Sequencing

Genomic DNA was extracted from the STEC isolates using the MagAttract HMW DNA Kit (Qiagen, Hilden, Germany) following the manufacturer’s protocol. Library preparation was performed using the MGIEasy FS DNA Library Prep Set (MGI Tech Co., Ltd., Shenzhen, China) in accordance with the manufacturer’s instructions. Briefly, 100 ng of genomic DNA was enzymatically fragmented and size-selected using magnetic beads. The fragmented DNA was subsequently end-repaired, ligated with adapters, and amplified through PCR (eight cycles) to generate the final library. The libraries were then loaded onto an MGI DNBSEQ G400 High-Throughput Sequencing Set FCL Flow Cell and sequenced on the DNBSEQ G400 RS platform (MGI Tech Co.) using paired-end reads of 150 bp × 2.

### 2.4. Whole-Genome Data Processing and Analysis

Raw sequencing reads were processed using fastp v0.19.5 for quality control, including adapter trimming and filtering of low-quality reads and adapter sequences [25]. Then, the filtered reads were assembled de novo using Unicycler v0.5.1 to generate draft genome assemblies [26]. Downstream analysis of the assembled genomes was conducted using tools hosted by the Center for Genomic Epidemiology (https://www.genomicepidemiology.org/ accessed on 20 January 2025). Specifically, antimicrobial resistance genes were identified using ResFinder v4.6.0, the serotype was determined using SeroTypeFinder v2.0, multilocus sequence types (MLST) were assigned using MLST v2.0, and VirulenceFinder v2.0 was used to detect virulence-associated genes based on the assembled genome sequences.

### 2.5. Phylogenetic Analysis

Comparative genome data were retrieved from the *Escherichia*/*Shigella* database in EnteroBase (https://enterobase.warwick.ac.uk/ accessed on 20 January 2025) [27]. All available assembly data for serotypes O38:H26 (*n* = 55) and O43:H2 (*n* = 68) were included in the analysis. For serotype O157:H7, representative genome assemblies (*n* = 50), including *E. coli* EDL933 reference strain (accession no. AE005174) [28], were selected from the data generated after 2020, ensuring diversity in source, country of origin, MLST, and the presence of *stx1* and *stx2* genes. Pan-genome analysis was performed using Roary v3.13.0 [29]. A maximum likelihood phylogenetic tree was constructed using IQ-TREE v2.3.0 [30], and the resulting tree was visualized with Interactive Tree of Life iTOL v7 [31].

## 3. Results

### 3.1. Detection Rates of STEC on Chromogenic Agar and Presence of stx Genes in Livestock and Companion Animals

Table 1 summarizes the isolation rates of STEC and the detection of *stx* genes in fecal samples from livestock and companion animals. Among the 97 samples analyzed, 17 (17.5%) tested positive for STEC chromogenic agar. All isolates demonstrated high sequence similarity to *E. coli* based on 16S rRNA gene DNA sequencing. Sheep exhibited the highest detection rate (27.3%, 9/33), followed by goats (18.2%, 6/33) and dogs (6.7%, 2/30). Screening for *stx* genes, conducted on isolates grown on STEC chromogenic agar, revealed that only samples from goats (12.1%, 4/33) and sheep (12.1%, 4/33) tested positive. Both *stx1* and *stx2* were co-detected in five isolates, one from a goat and four from sheep. In addition, one goat isolate exhibited *stx1*(+)/*stx2*(−), while two goat isolates were positive for *stx1*(−)/*stx2*(+).

### 3.2. Genetic and Phenotypic Characteristics of STEC Isolates

The eight STEC isolates recovered from goats and sheep were classified into three serotypes: O38:H26, O43:H2, and O157:H7 (Table 2). MLST analysis identified four sequence types (STs): ST10, ST11, ST11-like, and ST937 (Table 2). Among the eight STEC isolates, serotype O38:H26 was consistently associated with ST10, O43:H2 with ST937, and O157:H7 with either ST11 or ST11-like. No acquired antimicrobial resistance genes were identified in the STEC isolates based on ResFinder analysis. Furthermore, antimicrobial susceptibility testing demonstrated that all isolates were susceptible to the tested antimicrobial agents (Table 3). Virulence genes associated with enterohemorrhagic *E. coli* were detected in the isolates [32], with notable serotype-specific differences in gene profiles (Table 2). The *stx1c* gene was detected in all isolates of the O38:H26 and O43:H2 serotypes, while *stx2b* was identified in all O38:H26 isolates and in two of the three O43:H2 isolates. The *stx2c* gene was exclusively detected in the O157:H7 serotype. In addition, the *eae* gene, which is associated with the formation of attaching and effacing lesions, along with colonization-related genes, including *chuA*, *espA*, *espB*, *espJ*, *espP*, *espY2*, *nleA*, *nleB*, *nleC*, *tir*, and *toxB*, were present in all O157:H7 isolates. The colonization-related *espI* gene was identified in all O38:H26 isolates.

### 3.3. Phylogenetic Analysis of STEC Isolates

**Figure 1:**
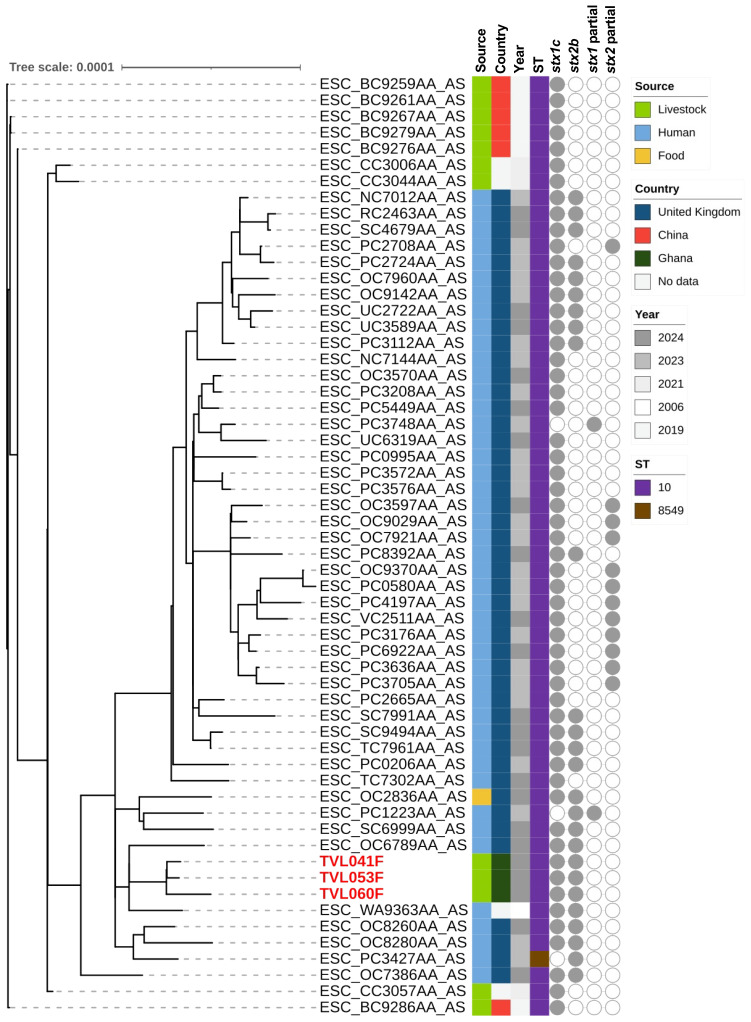
Maximum likelihood phylogenetic tree of *Escherichia coli* serotype O38:H26 genomes, including isolates from this study and publicly available data, with metadata on source, country, collection year, sequence type, and *stx* gene profiles. The isolates identified in the study are indicated by red-colored text.

**Figure 2:**
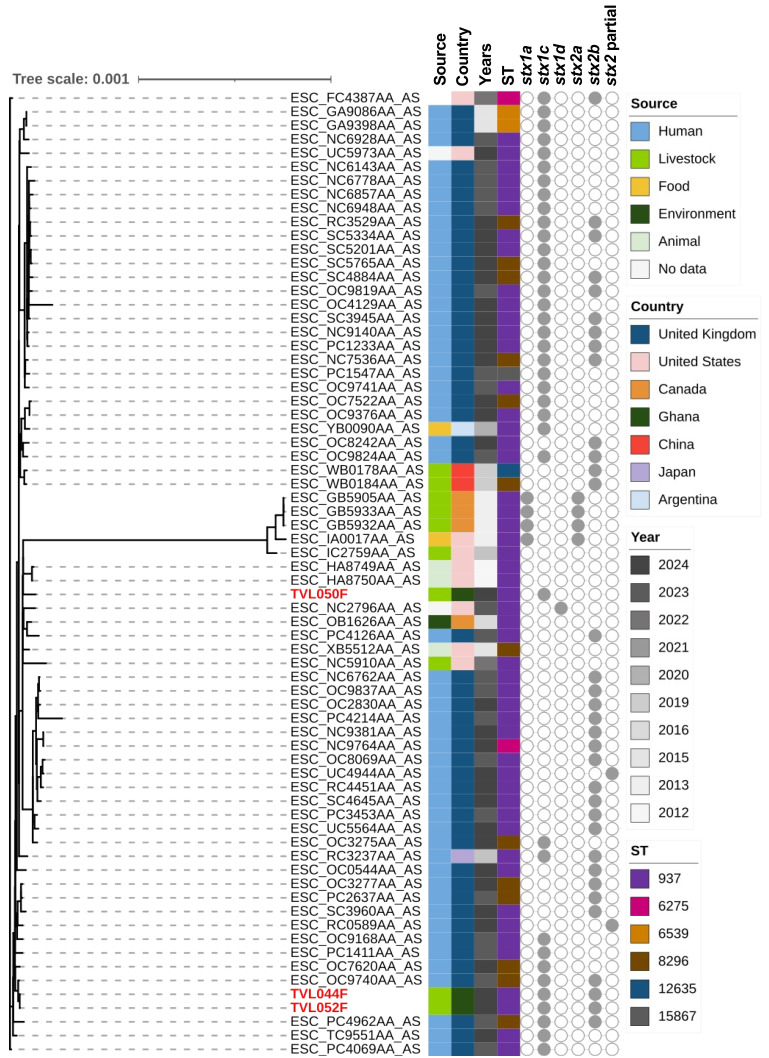
Maximum likelihood phylogenetic tree of *Escherichia coli* serotype O43:H2 genomes, including isolates from this study and publicly available data, with metadata on source, country, collection year, sequence type, and *stx* gene profiles. The isolates identified in the study are indicated by red-colored text.

**Figure 3:**
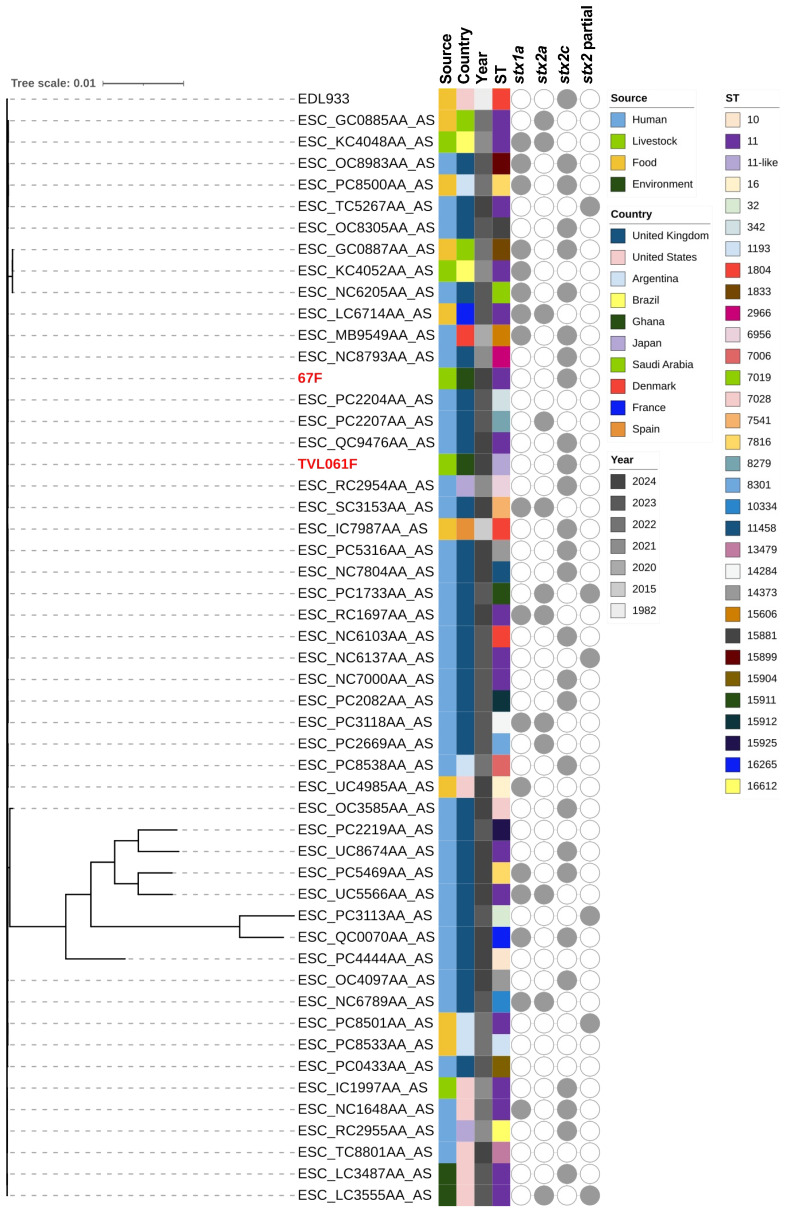
Maximum likelihood phylogenetic tree of *Escherichia coli* serotype O157:H7 genomes, including isolates from this study and publicly available data, with metadata on source, country, collection year, sequence type, and *stx* gene profiles. The isolates identified in the study are indicated by red-colored text.

Phylogenetic trees of the STEC isolates were constructed to investigate their genetic relationships with publicly available genomes of the same serotypes (O38:H26, O43:H2, and O157:H7; Supplementary Tables S1–S3). The phylogenetic analysis of the O38:H26 isolates revealed that all O38:H26 isolates were ST10, except for one identified as ST8549. The three isolates obtained from this study form a monophyletic clade (Figure 1), closely related to clinical isolates from the United Kingdom, sharing identical characteristics (ST10, *stx1c*, and *stx2b*). Among the publicly available O38:H26 genomes in the database, strains harboring either *stx1c* or *stx2b* alone were relatively common. Conversely, the co-occurrence of both genes, as identified in our isolates, was reported less frequently. For serotype O43:H2, the isolates displayed variability in their *stx* gene profiles, with different combinations of *stx1a*, *stx1c*, *stx1d*, *stx2a*, and *stx2b* genes (Figure 2). Specifically, TVL050F, which lacked the *stx2b* gene, formed a distinct cluster from TVL044F and TVL052F, both of which carried the *stx2b* gene. TVL050F was closely related to strains from animal, environmental, and clinical sources in North America, suggesting its ability for environmental dissemination. Conversely, TVL044F and TVL052F were more closely related to clinical isolates reported in the United Kingdom. A wide range of STs has been reported among O157:H7 isolates, with the *stx2c* gene being identified across diverse strains (Figure 3). To improve phylogenetic resolution and better visualize the subtle differences among closely related strains, we reconstructed the tree after excluding distantly related strains (Supplementary Figure S1). The isolates from this study were closely related to clinical and food-derived strains from Europe, emphasizing their possible association with human infections and foodborne transmission.

## 4. Discussion

In this study, STEC was not detected in companion animals; however, diverse serotypes of STEC were identified in goats and sheep, emphasizing their role as critical reservoirs. STEC has also been shown to persist more effectively in the alimentary tract of sheep compared to other *E. coli* pathotypes [33]. These results highlight the need for targeted surveillance and control strategies for small domestic ruminants to reduce the risk of STEC transmission to humans.

The epidemiology of STEC in Africa varies due to regional differences in livestock management practices, environmental conditions, and public health systems, with studies confirming its widespread distribution across the continent [34,35]. Various STEC serotypes have been identified in livestock animals, particularly goats, sheep, cattle, and camels, with reported prevalence rates ranging from 4.9% to 12.3%, depending on geographical region, including Ethiopia [17], Kenya [18], Nigeria [16], and South Africa [19]. In the present study, *stx*-positive STEC were detected in 12.1% of both goats and sheep, suggesting that the prevalence level of STEC in Ghana is comparable to those reported elsewhere in Africa and highlighting the widespread dissemination of STEC in the region. Moreover, *stx2*-positive STEC have been sporadically identified in outpatient children under five years of age in the Western Region of Ghana [1]. Another study from the Greater Accra Region of Ghana reported that 4.3% of preschool-aged children tested positive for STEC, with household ownership of goats or sheep significantly associated with STEC carriage in children [36]. These findings indicate a potential zoonotic transmission pathway, warranting further research to assess the epidemiological significance and public health implications of STEC.

Strains of O38:H26 and O43:H2 serotypes have been isolated from animals and animal-derived products in Asia, Europe, and Africa [37,38,39,40], indicating their widespread distribution in livestock and food sources. Notably, these serotypes have also been recovered from clinical samples in New Zealand and Romania [41,42]. The ST10 STEC, which was linked to the O38:H26 serotype in this study, was previously isolated from clinical specimens in Ghana, although the associated serotype had not been characterized, suggesting a localized dissemination pattern in the region [1]. The presence of *stx* genes in the O38:H26 and O43:H2 serotype strains isolated in this study, combined with their phylogenetic relatedness to clinical strains, suggests their potential pathogenicity and contribution to human infections, highlighting the importance of One Health-based surveillance strategies to monitor potential zoonotic transmission. Conversely, the O157:H7 serotype is globally recognized for its association with numerous outbreaks and its ability to cause severe STEC infections in humans [11,12]. Consistent with our findings, O157:H7 has been widely detected in livestock and exhibits close genetic relatedness to clinical isolates [11,12,16,17,43], highlighting its significant contribution to human transmission. In addition, the presence of key virulence genes, such as *stx2c* and *eae*, in O157:H7 isolates underscores their pathogenicity [6,44]. Furthermore, the detection of the *chuA*, *esp*, *nle*, *tir*, and *toxB* genes, which are associated with colonization in livestock and environmental adaptation, suggests their potential roles in the survival and persistence of STEC in diverse environments [45,46]. These results underscore the critical importance of systematically accumulating serotype-specific epidemiological data to assess pathogenic potential, elucidate transmission dynamics, and enhance targeted public health interventions.

This study has some limitations. Primarily, its small sample size and focus on a single geographic region may restrict the applicability of the findings to other areas in Ghana. Although cattle are recognized as the principal reservoir of STEC, they were not included in this study [4]. This limitation reflects the livestock production context in Ghana, where small ruminants, particularly goats and sheep, are commonly raised [47]. In addition, while the study sheds light on the genetic characteristics of STEC isolates from livestock, it does not address environmental transmission pathways, such as the potential spread of STEC through livestock fecal contamination of rivers and other water sources [43,48]. Detection of *E. coli* O157:H7 in 8% of water samples from 50 sites in Ethiopia highlights the role of contaminated water as a potential source of STEC transmission [43]. Future studies with larger sample sizes and broader geographic coverage are necessary to gain a better understanding of STEC epidemiology in Ghana.

This study presents the first report of STEC isolation from goats and sheep in Ghana, as well as the first whole-genome analysis of these isolates in the country. The presence of diverse serotypes and key virulence genes in these isolates from livestock suggests that they play a role as reservoirs for human infections. These findings underscore the importance of continued targeted surveillance and control efforts for STEC within the One Health framework, providing essential data to inform public health initiatives in Ghana.

## Figures and Tables

**Table 1:** Positive rates of Shiga toxin-producing *Escherichia coli* from livestock and companion animals.

Species	Number of Sample	STEC Chromogenic Agar	*stx1*(+)/*stx2*(+)	*stx1*(+)/*stx2*(−)	*stx1*(−)/*stx2*(+)
Goat	33	6 (18.2%)	1 (3.0%)	1 (3.0%)	2 (6.1%)
Sheep	33	9 (27.3%)	4 (12.1%)	0	0
Dog	30	2 (6.7%)	0	0	0
Cat	1	0	0	0	0
All	97	17 (17.5%)	5 (5.2%)	1 (1.0%)	2 (2.1%)

**Table 2:** Serotype and virulence profile of Shiga toxin-producing *Escherichia coli*.

Sample	TVL041F	TVL044F	TVL050F	TVL052F	TVL053F	TVL060F	TVL061F	67F
Species	Sheep	Goat	Goat	Sheep	Sheep	Sheep	Goat	Goat
Serotype	O38:H26	O43:H2	O43:H2	O43:H2	O38:H26	O38:H26	O157:H7	O157:H7
Sequence type	ST10	ST937	ST937	ST937	ST10	ST10	ST11	ST11-like
Virulence genes linked to enterohemorrhagic *E. coli*								
*chuA*	**-**	**-**	**-**	**-**	**-**	**-**	**+**	**+**
*eae*	**-**	**-**	**-**	**-**	**-**	**-**	**+**	**+**
*espA*	**-**	**-**	**-**	**-**	**-**	**-**	**+**	**+**
*espB*	**-**	**-**	**-**	**-**	**-**	**-**	**+**	**+**
*espI*	**+**	**-**	**-**	**-**	**+**	**+**	**-**	**-**
*espJ*	**-**	**-**	**-**	**-**	**-**	**-**	**+**	**+**
*espP*	**-**	**-**	**-**	**-**	**-**	**-**	**+**	**+**
*espY2*	**-**	**-**	**-**	**-**	**-**	**-**	**+**	**+**
*nleA*	**-**	**-**	**-**	**-**	**-**	**-**	**+**	**+**
*nleB*	**-**	**-**	**-**	**-**	**-**	**-**	**+**	**+**
*nleC*	**-**	**-**	**-**	**-**	**-**	**-**	**+**	**+**
*stx1c*	**+**	**+**	**+**	**+**	**+**	**+**	**-**	**-**
*stx2b*	**+**	**+**	**-**	**+**	**+**	**+**	**-**	**-**
*stx2c*	**-**	**-**	**-**	**-**	**-**	**-**	**+**	**+**
*tir*	**-**	**-**	**-**	**-**	**-**	**-**	**+**	**+**
*toxB*	**-**	**-**	**-**	**-**	**-**	**-**	**+**	**+**

**Table 3:** Susceptibility profile of Shiga toxin-producing *Escherichia coli*.

Antibiotics	TVL041F	TVL044F	TVL050F	TVL052F	TVL053F	TVL060F	TVL061F	67F
Ampicillin	≦8 (S)	≦8 (S)	≦8 (S)	≦8 (S)	≦8 (S)	≦8 (S)	≦8 (S)	≦8 (S)
Ampicillin- sulbactam	≦8/4 (S)	≦8/4 (S)	≦8/4 (S)	≦8/4 (S)	≦8/4 (S)	≦8/4 (S)	≦8/4 (S)	≦8/4 (S)
Ceftolozane- tazobactam	≦2/4 (S)	≦2/4 (S)	≦2/4 (S)	≦2/4 (S)	≦2/4 (S)	≦2/4 (S)	≦2/4 (S)	≦2/4 (S)
Piperacillin- tazobactam	≦8/4 (S)	≦8/4 (S)	≦8/4 (S)	≦8/4 (S)	≦8/4 (S)	≦8/4 (S)	≦8/4 (S)	≦8/4 (S)
Cefazolin	≦2 (S)	≦2 (S)	≦2 (S)	≦2 (S)	≦2 (S)	≦2 (S)	≦2 (S)	≦2 (S)
Cefepime	≦2 (S)	≦2 (S)	≦2 (S)	≦2 (S)	≦2 (S)	≦2 (S)	≦2 (S)	≦2 (S)
Ceftriaxone	≦1 (S)	≦1 (S)	≦1 (S)	≦1 (S)	≦1 (S)	≦1 (S)	≦1 (S)	≦1 (S)
Ceftadizime	≦1 (S)	≦1 (S)	≦1 (S)	≦1 (S)	≦1 (S)	≦1 (S)	≦1 (S)	≦1 (S)
Cefmetazole	≦16 (S)	≦16 (S)	≦16 (S)	≦16 (S)	≦16 (S)	≦16 (S)	≦16 (S)	≦16 (S)
Cefpodoxime	≦1 (S)	≦1 (S)	≦1 (S)	≦1 (S)	≦1 (S)	≦1 (S)	≦1 (S)	≦1 (S)
Cefpodoxime- clavulanate	≦1	≦1	≦1	≦1	≦1	≦1	≦1	≦1
Cefditoren	≦1	≦1	≦1	≦1	≦1	≦1	≦1	≦1
Flomoxef	≦8	≦8	≦8	≦8	≦8	≦8	≦8	≦8
Latamoxef	≦8	≦8	≦8	≦8	≦8	≦8	≦8	≦8
Aztreonam	≦4 (S)	≦4 (S)	≦4 (S)	≦4 (S)	≦4 (S)	≦4 (S)	≦4 (S)	≦4 (S)
Meropenem	≦0.12 (S)	≦0.12 (S)	≦0.12 (S)	≦0.12 (S)	≦0.12 (S)	≦0.12 (S)	≦0.12 (S)	≦0.12 (S)
Faropenem	≦2	≦2	≦2	≦2	≦2	≦2	≦2	≦2
Colistin	≦1 (I)	≦1 (I)	≦1 (I)	≦1 (I)	≦1 (I)	≦1 (I)	≦1 (I)	≦1 (I)
Gentamicin	≦4 (S)	≦4 (S)	≦4 (S)	≦4 (S)	≦4 (S)	≦4 (S)	≦4 (S)	≦4 (S)
Amikacin	≦16 (S)	≦16 (S)	≦16 (S)	≦16 (S)	≦16 (S)	≦16 (S)	≦16 (S)	≦16 (S)
Minocycline	≦4 (S)	≦4 (S)	≦4 (S)	≦4 (S)	≦4 (S)	≦4 (S)	≦4 (S)	≦4 (S)
Levofloxacin	≦0.12 (S)	≦0.12 (S)	≦0.12 (S)	≦0.12 (S)	≦0.12 (S)	≦0.12 (S)	≦0.12 (S)	≦0.12 (S)
Trimethoprim- sulfamethoxazole	≦2/38 (S)	≦2/38 (S)	≦2/38 (S)	≦2/38 (S)	≦2/38 (S)	≦2/38 (S)	≦2/38 (S)	≦2/38 (S)
Fosfomycin	≦4 (S)	≦4 (S)	≦4 (S)	≦4 (S)	≦4 (S)	≦4 (S)	≦4 (S)	≦4 (S)
Tigecycline	≦1	≦1	≦1	≦1	≦1	≦1	≦1	≦1

Antibiotic susceptibility is shown with interpretation in parentheses: S (susceptible), I (intermediate), R (resistant).

## Data Availability

The data presented in this study are openly available in the NCBI database under reference number(s): SAMN47554130 (TVL041F), SAMN47554131 (TVL044F), SAMN47554133 (TVL050F), SAMN47554134 (TVL052F), SAMN47554136 (TVL053F), SAMN47554137 (TVL060F), SAMN47554139 (TVL061F), SAMN47554142 (67F).

## Supplementary Materials

The following supporting information can be downloaded at: https://www.mdpi.com/article/10.3390/microorganisms14010212/s1, Figure S1: Maximum likelihood phylogenetic tree of *Escherichia coli* serotype O157:H7 genomes by excluding distantly related strains, with metadata on source, country, collection year, sequence type, and *stx* gene profiles; Table S1: Metadata of O38:H26 isolates used for phylogenetic analysis; Table S2: Metadata of O43:H2 isolates used for phylogenetic analysis; Table S3: Metadata of O157:H7 isolates used for phylogenetic analysis.

## Abbreviations

The following abbreviations are used in this manuscript:

STEC	Shiga toxin-producing *Escherichia coli*
*E. coli*	*Escherichia coli*
Stx	Shiga toxin
HUS	Hemolytic uremic syndrome
MLST	Multilocus sequence typing
ST	Sequence type
