# Supplementary material for: Whole-Genome Sequence Analysis of Shiga Toxin-Producing Escherichia coli Isolated from Livestock Animals in Ghana
Source: Microorganisms. 2026 Jan 16;14(1):212. doi: 10.3390/microorganisms14010212 (PMC12844413; doi:10.3390/microorganisms14010212)
Supplement: Supplementary file 1 [file microorganisms-14-00212-s001.zip › Supplementary Figure.pdf]

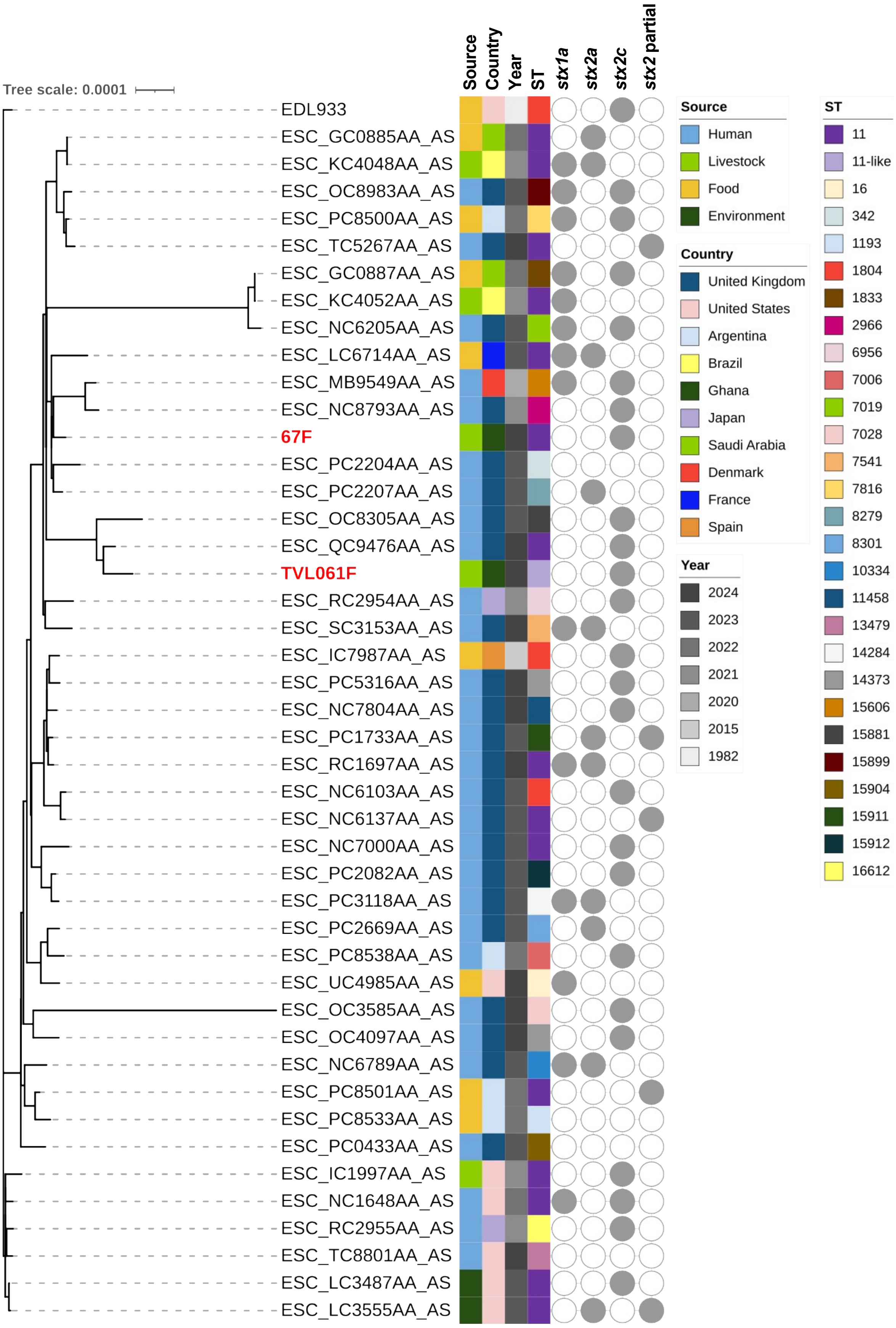

Tree scale: 0.0001
Source
Country
Year
ST
stx1a
stx2a
stx2c
stx2 partial
EDL933
ESC_GC0885AA_AS
ESC_KC4048AA_AS
ESC_OC8983AA_AS
ESC_PC8500AA_AS
ESC_TC5267AA_AS
ESC_GC0887AA_AS
ESC_KC4052AA_AS
ESC_NC6205AA_AS
ESC_LC6714AA_AS
ESC_MB9549AA_AS
ESC_NC8793AA_AS
67F
ESC_PC2204AA_AS
ESC_PC2207AA_AS
ESC_OC8305AA_AS
ESC_QC9476AA_AS
TVL061F
ESC_RC2954AA_AS
ESC_SC3153AA_AS
ESC_IC7987AA_AS
ESC_PC5316AA_AS
ESC_NC7804AA_AS
ESC_PC1733AA_AS
ESC_RC1697AA_AS
ESC_NC6103AA_AS
ESC_NC6137AA_AS
ESC_NC7000AA_AS
ESC_PC2082AA_AS
ESC_PC3118AA_AS
ESC_PC2669AA_AS
ESC_PC8538AA_AS
ESC_UC4985AA_AS
ESC_OC3585AA_AS
ESC_OC4097AA_AS
ESC_NC6789AA_AS
ESC_PC8501AA_AS
ESC_PC8533AA_AS
ESC_PC0433AA_AS
ESC_IC1997AA_AS
ESC_NC1648AA_AS
ESC_RC2955AA_AS
ESC_TC8801AA_AS
ESC_LC3487AA_AS
ESC_LC3555AA_AS
Source
Human
Livestock
Food
Environment
Country
United Kingdom
United States
Argentina
Brazil
Ghana
Japan
Saudi Arabia
Denmark
France
Spain
Year
2024
2023
2022
2021
2020
2015
1982
ST
11
11-like
16
342
1193
1804
1833
2966
6956
7006
7019
7028
7541
7816
8279
8301
10334
11458
13479
14284
14373
15606
15881
15899
15904
15911
15912
16612

Supplementary Figure S1. Maximum likelihood phylogenetic tree of *Escherichia coli* serotype O157:H7 genomes by excluding distantly related strains, with metadata on source, country, collection year, sequence type, and *stx* gene profiles.
